# Differences Regarding Knowledge of Sexually Transmitted Infections, Sexual Habits, and Behavior Between University Students of Medical and Nonmedical Professions in Serbia

**DOI:** 10.3389/fpubh.2021.692461

**Published:** 2022-01-13

**Authors:** Slobodan Subotic, Vladimir Vukomanovic, Svetlana Djukic, Svetlana Radevic, Snezana Radovanovic, Danijela Radulovic, Katarina Boricic, Jelena Andjelkovic, Jelena Tosic Pajic, Ivana Simic Vukomanovic

**Affiliations:** ^1^College of Health Studies Milutin Milankovic, Belgrade, Serbia; ^2^Department of Nuclear Medicine, Faculty of Medical Sciences, University of Kragujevac, Kragujevac, Serbia; ^3^Department of Internal Medicine, Faculty of Medical Sciences, University of Kragujevac, Kragujevac, Serbia; ^4^Department of Social Medicine, Faculty of Medical Sciences, University of Kragujevac, Kragujevac, Serbia; ^5^Department of Primary Health Care and Public Health, Faculty of Medicine, University of East Sarajevo, Sarajevo, Bosnia and Herzegovina; ^6^Dr. Milan Jovanovic Batut Institute of Public Health of Serbia, Belgrade, Serbia; ^7^Medicines and Medical Devices Agency of Serbia (ALIMS), Belgrade, Serbia; ^8^Clinical Centre of Kragujevac, Kragujevac, Serbia

**Keywords:** students, sexually transmitted infections, knowledge, sexual behavior, reproductive health

## Abstract

**Aim:** The aim of this study is to assess the knowledge of sexually transmitted infections (STIs), sexual habits, and behavior among students of medical and nonmedical students in Serbia.

**Methodology:** The cross-sectional study of 1,273 university students of four undergraduate institutions in Serbia, two of medical and two of nonmedical orientation. A standardized questionnaire, prepared in line with the questionnaire of the European health research—the second wave (European Health Interview Survey—EHIS wave 2), according to defined internationally accepted indicators, was used as a survey instrument.

**Results:** Statistically significant difference (*p* < 0.001) between medical and nonmedical student groups was determined for the following parameters: naming four of five STIs (29.1 vs. 13.4%), knowledge about vaccines against some STIs (26.0 vs. 17.0%), relationship between HPV infection and cervical malignancy (48.2 vs. 16.7%) engaged in the sexual relations (87.9 vs. 76.4%), never used a condom (15.2 vs. 10.4%), underwent gynecological or urological examination (66.7 vs. 44.1%), and tested to one of STIs (10.5 vs. 4.9%).

**Conclusion:** Both student groups have limited knowledge on possible consequences that risky sexual behavior has for reproductive health. Promotion of knowledge about STIs, awareness of all complications, and consequences of these infections certainly affect the reduction of risky behavior.

## Introduction

The term sexually transmitted infections (STIs) include a range of clinical syndromes caused by various types of pathogens that may be transmitted through sexual activity. STIs represent significant public health issues that may lead to acute diseases, long-term health complications, infertility, and premature death (1). According to the reports released by the World Health Organization (WHO), the number of cases of STIs is constantly increasing, and today, they account for 376.4 million new cases worldwide (2).

Young people are mostly at risk from STIs, although they represent only 25% of the sexually active population. According to the latest data, young people aged 15–24 years represent almost 50% of all newly acquired STIs worldwide (3–5). Additionally, it is the period characterized by self-identity research, followed by young people's reaching for autonomy, and also engaging in sexual activity (6).

Numerous research of sexual behavior among university students shows that students are a high-risk population which is at an increased risk of acquiring and transmitting STIs (7). Risky sexual behavior of students includes frequent partner change, sexual activity under the influence of psychoactive substances, and inconsistent condom usage, etc. (8, 9). The literature has shown that there is a number of factors contributing to risky sexual behaviors among university students. It was observed that childhood abuse, poor mental health, alcohol use, drug use, partner violence, or sexual coercion are significantly correlated with risky sexual behaviors (10). Generally, students tend to experiment and are more likely to practice risky sexual behaviors due to their new sense of freedom felt at boarding institutions, which is also followed by the fact that they experience a sense of liberty from the familiarized community, their parents, and professors. University students are categorized under the most at-risk population segment due to their inclination to be engaged in risky sexual behaviors and their poor sense of vulnerability. Despite this, youth have not traditionally been considered a health priority since they have lower morbidity and mortality rates than older and younger age groups (11). The lack of knowledge regarding sexually transmitted diseases (STDs) among college students is a significant problem not only because this is the age group mostly at risk for acquiring an STD, but also because irreversible damage can be caused by STDs if not treated immediately, that is, it can lead to infertility (12).

For the purpose of STD prevention, there are HIV/AIDS/STD counseling centers at the Public Health Institutes on the territory of the Republic of Serbia. These counseling centers base their work upon confidential, voluntary counseling and testing and are available to the youth population on a daily basis. Moreover, for the purpose of cervical cancer prevention, there is the National Cervical Cancer Early Detection Program (a Pap test), which is administered on the territory of the Republic of Serbia. The target population are women between the age of 25 and 64 years. The results of the abovementioned National Program indicate not only insufficient levels of knowledge of these women about the efficiency of cervical cancer preventive measures, but also a low range of the targeted female population who undergo regular Pap testing and screening as well (13).

It is considered that higher levels of knowledge about sexual health provide many benefits to these categories. The promotion of knowledge about STIs completes with raising the level of awareness of all potential complications and consequences associated with the abovementioned infections and certainly plays a significant role in reducing risky behaviors (6). Having good knowledge of STIs is one of the protective factors necessary for students, so that they could become aware of the modes of STI transmission, preventive measures, and its complications, which may eventually help them to take care of themselves and lessen the risk of infection for all STIs (11). The aim of this study is to assess the knowledge of STIs, sexual habits, and behaviors among medical and nonmedical students.

## Materials and Methods

### Type of Study and Population

The study was designed as a cross-sectional study of students who attended the University of Belgrade, Serbia, in the year 2017–2018. The study population included students from four higher education institutions. The students who attended all 3 years of undergraduate studies, from different educational profiles, were included in the study. The first two undergraduate institutions were in the field of medical sciences (College for Health Studies “Milutin Milankovic” and College for Health Studies, University of Belgrade), and the other two were in the field of nonmedical sciences (Business Academy and the Faculty of Security Studies, University of Belgrade).

### Sampling

The students were randomly sampled from every study year of selected faculties, in proportion to the size of the faculty. From the total number of 1,353 students, 1,273 accepted to fill the questionnaire (the response rate of 94.1%). After the approval had been granted by the Dean of the selected faculties, the study was conducted in the participants' own classrooms by the leading researcher (at the scheduled date and time agreed in advance).

The standardized questionnaire, prepared in line with the questionnaire of the European health research—the second wave (the European Health Interview Survey—EHIS wave 2), according to defined internationally accepted indicators, was used as a survey instrument.

The questionnaire in this study included three sections. The first section referred to the following participants' demographic characteristics: sex, age, place of residence, year of study, relationship status, education, smoking habit, and alcohol consumption. The second section of the questionnaire referred to the level of knowledge about STIs, such as the manner of acquiring knowledge in relation to STIs, STI transmission routes, STI types, symptoms related to STIs, STI prevention, and relationship between STIs and cervical cancer.

All the participants provided oral consent to the study participation and the anonymity criterion was met. The authorization for conducting the research was acquired from the Ethics Committee of undergraduate institutions of the University, and it was conducted in accordance with the Declaration of Helsinki.

### Statistical Analysis

The complete statistical analysis was performed using the statistical software package, PASW Statistics 18® [SPSS (Hong Kong) Ltd., Hong Kong]. All variables were presented as the frequency of certain categories. Differences between categorical variables were tested by the chi-square test, whereas the significance of differences between continuous variables was tested by the nonparametric Mann–Whitney *U*-test. The normality of the data was assessed using the Kolmogorov–Smirnov test. The relationship between the variables was tested by Spearman's rank correlation coefficient. All the analyses were estimated at *p* < 0.05 significance level.

## Results

### Sociodemographic Characteristics of the Student Population

The students were approximately 67% of women in both analyzed groups (66.8% in the group with medical education and 67.0% in the group with nonmedical education). However, there was a statistically significant difference in relation to student's age (*p* < 0.001). The median age was 1 year higher in the medical student group in comparison with the nonmedical student group (age of 22 vs. 21 years). When students were divided into two age groups, it could be observed that students in the age range of 21–30 years dominated in both analyzed groups, whereas there were significantly few students in other groups.

Regarding the relationship status, 46.2% of the medical students were in a relationship, whereas 49.0% of the nonmedical students were single (Table 1).

**Table 1:** Sociodemographic characteristics of study population.

**Variables**	**Students of medical profession no = 645**	**Nonmedical students no = 628**	***p*-value**
**Sex**			
Male	214 (33.2)	207 (33.0)	0.982^*^
Female	431 (66.8)	421 (67.0)	
**Age**	22 (20-27)	21 (20-21)	<0.001^**^
<20	184 (28.5)	305 (48.6)	<0.001^*^
21–30	343 (53.2)	317 (50.5)	
31–40	90 (14.0)	5 (0.8)	
>41	28 (4.3)	1 (0.2)	
**Study year**			
First	227 (35.2)	230 (36.6)	<0.375^*^
Second	307 (47.6)	276 (43.9)	
third	111 (17.2)	122 (19.4)	
**Relationship status**			
Married	121 (18.8)	19 (3.0)	<0.001^*^
In a relationship	298 (46.2)	294 (46.9)	
Divorced	13 (2.0)	7 (1.1)	
Single	212 (32.9)	308 (49.0)	

* *Chi-square test*;

** *Mann-Whitney U-test; No, number*.

### The Level of Student's Knowledge on STIs

There was a significant difference observed between these student groups in relation to the manner of acquiring knowledge on STIs (*p* < 0.001). The medical students mostly acquired knowledge at school or college (86.0%) and *via* the Internet (59.4%), whereas the nonmedical students mostly acquired knowledge in the Counsel for Youth (89.0%), from their family (59.5%), friends (54.6%), daily newspapers (79.4%), or in some other manner (94.3%).

The majority of medical students answered the question that required them to list five STIs by naming four of five (29.1 vs. 13.4%; *p* < 0.001) STDs, whereas most of the nonmedical students, 32.6% of them, could not name a single STD. On average, the medical students named 3.07 ± 1.69 different diseases, whereas the nonmedical students named 1.95 ± 1.76 diseases, which represented a statistically significant difference (*p* < 0.001).

Having analyzed different types of STDs that students named, there were significant differences observed in most categories, which implies that there was a vast difference in the type of STDs named by the students from the two groups (*p* < 0.001). The medical students most frequently listed HIV (82.6%), syphilis (66.4%), and gonorrhea (46.8%), whereas the nonmedical students most frequently listed genital herpes (95.2%), candida (96.7%), and chlamydia (87.3%.)

A significant number of students from the nonmedical group did not know the answer to the question of whether STIs always have pronounced symptoms (30.4 vs. 15.4%; *p* < 0.001). Additionally, there was a significant difference noticed between the two groups related to their respond affirmatively to this particular question. The medical students showed a higher level of knowledge and they gave positive responses (62.6 vs. 49.7%; *p* < 0.001).

There was no significant difference observed in the level of knowledge of the STI transmission through sharing needles and syringes. In both groups, the students responded affirmatively to the question related to the possibility of STI transmission through sharing needles and syringes (78.6 vs. 77.5%; *p* = 0.130). A certain number of students in both groups did not know the answer to this question (10.1 vs.13.2%; *p* = 0.130.).

Concerning the knowledge about preventive measures, such as vaccines against some STIs, the medical students more commonly provided an affirmative answer (26.0 vs. 17.0%; *p* < 0.001). Consequently, the medical students more commonly provided the answer that there was a relationship between HPV infection and cervical malignancy (48.2 vs. 16.7%; *p* < 0.001; Table 2).

**Table 2:** Student's knowledge about STIs.

**Variables**	**Students of medical profession no = 645**	**Nonmedical students no = 628**	***p*-value**
**WHERE THE KNOWLEDGE ON STI WAS ACQUIRED:**			
Family	287 (44.5)	373 (59.5)	
Friends	254 (39.4)	343 (54.6)	
School/college	555 (86.0)	296 (47.1)	
Counsel for youth	80 (12.4)	559 (89.0)	<0.001^*^
Community health center	123 (19.1)	536 (85.4)	
Internet	383 (59.4)	278 (44.3)	
Daily newspapers	191 (29.6)	498 (79.4)	
Other	6 (0.9)	592 (94.3)	
**NUMBER OF STI TYPES LISTED:**			
None	95 (14.7)	205 (32.6)	<0.001^*^
1	40 (6.2)	88 (14.0)	
2	62 (9.6)	86 (13.7)	
3	117 (18.1)	97 (15.4)	
4	188 (29.1)	84 (13.4)	
5	143 (22.2)	68 (10.8)	
**STI TYPES:**			
HIV	533 (82.6)	220 (35.0)	<0.001^*^
Syphilis	428 (66.4)	409 (65.1)	
Gonorrhea	302 (46.8)	465 (74.0)	
Chlamydia	80 (12.4)	548 (87.3)	
Genital herpes	47 (7.3)	598 (95.2)	
Candida	51 (7.9)	607 (96.7)	
**DOES STI ALWAYS HAS PRONOUNCED SYMPTOMS?**			
Yes	142 (22.0)	125 (19.9)	<0.001^*^
No	404 (62.6)	312 (49.7)	
I do not know	99 (15.4)	191 (30.4)	
**STI TRANSFER THROUGH NEEDLE?**			
Yes	507 (78.6)	487 (77.5)	0.130^*^
No	73 (11.3)	58 (9.2)	
I do not know	65 (10.1)	83 (13.2)	
**EVERY ORAL INTERCOURSE WITHOUT CONDOM IS RISKY?**			
Yes	359 (55.7)	286 (45.5)	<0.001^*^
No	286 (44.3)	342 (54.5)	
**IS THERE AN EFFECTIVE VACCINE AGAINST SOME STI?**			
Yes	168 (26.0)	107 (17.0)	<0.001^*^
No	224 (34.7)	114 (18.2)	
I do not know	253 (39.2)	407 (64.8)	
**IS THERE A RELATIONSHIP BETWEEN HPV INFECTION AND CERVICAL MALIGNANCY?**			
Yes	311 (48.2)	103 (16.4)	<0.001^*^
No	29 (4.5)	17 (2.7)	
I do not know	305 (47.3)	508 (80.9)	

* *Chi-square test; No, number; STIs, sexually transmitted infections*.

### Sexual Habits of the Student Population

There was a statistically significant difference found between the medical students and nonmedical students when comparing the answers they provided to the question of whether they had sexual relations or not. The medical students were significantly more frequently engaged in sexual relations than the nonmedical students (87.9 vs. 76.4%; *p* < 0.001). Regarding the students who were engaged in sexual relations, the medical students had their first sexual intercourse at the age of 18 (14–16) (the median with interquartile range), whereas the nonmedical students had their first sexual intercourse at the age of 18 (14, 15). As regards having sexual relations for the first time, the median age was the same for both study groups. However, based on its range, it could be observed that the interquartile range was wider for the medical students.

Regarding the number of partners in their lifetime, there was a statistically significant difference observed: the medical students more frequently changed their sexual partners [medical education, 2 (1–5) partners, nonmedical education, 1 (1–3) partner] (*p* < 0.001).

When comparing the frequency of sexual relations, there was a significant difference observed between the two groups (*p* < 0.001). Sexual relations were most commonly practiced 2–3 times a week, followed with 2–3 times a month. However, there were significantly more nonmedical students who never had sexual relations (12.1 vs. 22.6%; *p* < 0.001).

Regarding the condom usage during vaginal intercourse, 15.2% of the medical students and 10.4% of the nonmedical students never used a condom during vaginal intercourse. During oral intercourse, there was a greater number of the nonmedical students who never used a condom (27.2 vs. 19.7%; *p* < 0.001).

The most common reasons for not using a condom in both groups were related to the fact that they either trusted their partners (43.2 vs. 42.6%; *p* < 0.001) or that a condom reduced the sense of pleasure felt during sexual intercourse (25.7 vs. 30.1%; *p* < 0.001).

Regarding the use of contraceptive methods, the cumulative percentage frequency data were shown in the paper, since the students were able to choose more than one answer provided. Condoms were most often used in both groups, by more than 40% of the participants. The medical students more frequently used birth control pills (12.1% vs. 6.2%; *p* < 0.001) and *coitus interruptus* (the pull-out method) (15.9 vs. 7.8%; *p* < 0.001) than the nonmedical students. More students from the nonmedical group reported that they were virgins, whereas those who did not use any form of contraception were more common among the medical students. The medical students significantly more often reported going to the gynecologist or urologist in comparison with the nonmedical students (51.2 vs. 35.5%; *p* < 0.001). Over the past 3 years, 61.7% of the medical students were examined by doctors, which was significantly more when compared to 44.1% of the nonmedical students. Even 45.2% of the nonmedical students were never examined, which was significantly more when compared to 31.9% of the medical students.

In comparison with the nonmedical students, the medical students significantly more often underwent testing for STDs (10.5 vs. 4.9%; *p* < 0.001). In comparison with the medical students, the nonmedical students significantly more often reported to believe that they were not at risk of becoming infected with some STDs (52.1 vs. 61.8%; *p* < 0.002). It implied that a greater number of the medical students believed that they were at risk for acquiring some STDs (Table 3).

**Table 3:** Sexual habits of students and their sexual experience.

**Variables**	**Students of medical profession no = 645**	**Nonmedical students no = 628**	***p*-value**
Did you ever have sexual relations?	567 (87.9)	423 (76.4)	<0.001^*^
Age at first sexual relation	18 (17± 19)	18 (17± 18)	0.009^**^
Number of sexual partners over lifetime?	2 (1–5)	1 (1–3)	<0.001^**^
**PRACTICING SEXUAL RELATIONS**			
Every day	48 (7.4)	49 (7.8)	<0.001^*^
2–3 times a week	290 (45.0)	247 (39.3)	
1–3 times a month	170 (26.4)	117 (18.6)	
Once every 3 months	35 (5.4)	40 (6.4)	
Once every 6 months	9 (1.4)	20 (3.2)	
Once a year	15 (2.3)	13 (2.1)	
Never	78 (12.1)	142 (22.6)	
**CONDOM USE DURING VAGINAL SEXUAL INTERCOURSE**			
Always	164 (25.4)	172 (27.4)	<0.001^*^
Sometimes	300 (46.5)	239 (38.1)	
Never	98 (15.2)	65 (10.4)	
Virgin	78 (12.1)	149 (23.7)	
I do not practice	5 (0.8)	3 (0.5)	
**CONDOM USE DURING ORAL SEXUAL INTERCOURSE**			
Always	17 (2.6)	12 (1.9)	<0.001^*^
Sometimes	28 (4.3)	44 (7.0)	
Never	127 (19.7)	171 (27.2)	
Virgin	78 (12.1)	142 (22.6)	
I do not practice	395 (61.3)	259 (41.2)	
**REASON FOR NOT USING CONDOMS**			
They are expensive	19 (4.7)	28 (9.0)	<0.001^*^
Partner refuses to use them	17 (4.2)	20 (6.4)	
I am using other contraceptive measures	71 (17.5)	18 (5.8)	
I do not think that condom should be used	19 (4.7)	19 (6.1)	
I trust my partner	175 (43.2)	133 (42.6)	
It reduces pleasure during intercourse	104 (25.7)	94 (30.1)	
**CONTRACEPTIVE MEASURES**			
Birth control pills	78 (12.1)	39 (6.2)	<0.001^*^
Condoms	283 (43.9)	296 (47.1)	
Pull-out method	102 (15.9)	49 (7.8)	
Other	2 (0.3)	6 (1.0)	
Virgin	78 (12.1)	149 (23.7)	
I do not use them	110 (17.1)	89 (14.2)	
**LAST GYNECOLOGICAL OR UROLOGICAL EXAMINATION**			
I do not remember when	41 (6.4)	67 (10.7)	<0.001^*^
Never	206 (31.9)	284 (45.2)	
Over the past year	330 (51.2)	223 (35.5)	
Over the past three years	68 (10.5)	54 (8.6)	
**SEXUAL RELATIONS ON THE FIRST DATE**			
Yes	95 (14.7)	87 (13.9)	0.714^*^
No	550 (85.3)	541 (86.1)	
**I HAVE SEXUAL RELATIONS WHILE AFFECTED BY ALCOHOL OR OTHER PSYCHOACTIVE SUBSTANCES**			
Never	456 (70.7)	455 (72.5)	0.059^*^
Rarely	178 (27.6)	151 (24.0)	
Always	11 (1.7)	22 (3.5)	
**HAVE YOU BEEN TESTED TO ANY STI?**			
Yes	68 (10.5)	31 (4.9)	<0.001^*^
No	577 (89.5)	597 (95.1)	
**DO YOU THINK THAT THERE IS A RISK FOR YOU TO GET INFECTED BY ANY SEXUALLY TRANSMITTED DISEASE?**			
There is no risk	336 (52.1)	388 (61.8)	0.002^*^
There is a risk	129 (20.0)	103 (16.4)	
Moderate risk	180 (27.9)	137 (21.8)	

* *Chi-square test*;

** *Mann–Whitney U-test; No, number; STIs, sexually transmitted infections*.

## Discussion

The fact that the highest rates of STIs are being registered in young men and women is raising some concerns, taking into consideration that these infections can seriously deteriorate both male and female reproductive health.

As regards the demographic characteristics of participants in our study, there was a significantly larger number of female participants (67%), which was in accordance with the results of studies performed in Portugal (64%) and Laos (61.6%) (17, 18). In our study, participants (medical students) were older (age of 21–30 years) (53.2 vs. 50.5%), which was the case with the medical students in China (the median age ranges from 23 to 29 years—(25 years) (19).

The medical students in China reported being mostly single (67.15%), which differed from our medical students who were mostly in a relationship (46.2%) (19).

A detailed analysis of our results led us to the conclusion that 86% of the medical students gained most of their knowledge in colleges and schools. The results of the study which included 645 students at the medical universities in Serbia were in accordance with our results: male students (80.0%) and female students (88.6%) (14).

Nowadays, plenty of information related to this particular topic is easily available *via* the Internet data services. This also happened to be the case with the students in Malaysia (77.3%) (15), and medical students in Laos as well (76.6%), who gained most of their knowledge on the Internet (18). The nonmedical students in our study acquired knowledge from daily newspapers (79.4%), which was also the case with the Turkish students, who acquired knowledge from newspapers, magazines, and books as well (16). As regards sexual knowledge acquisition through parents or relatives, 59.5% of the nonmedical students in our study gave priority to their families (in comparison with 888 students from Turkey (59.5 vs.10.7%) (16). The results of the study that conducted based on the group of nonmedical students in Albania demonstrated that the Albanian students obtained information on sexual matter mostly from their teachers (49%) and parents (44%) (20), but the group of Sicilian students' and the group of Turkish students' most frequently referred sources of sexual health information were the Internet and television (16).

In Serbia, a lot of young people have poor knowledge of the anatomy and physiology of the male and female reproductive organs (21). Additionally, the knowledge on STIs among university students is scarce and burdened with a number of prejudices (22). One of the most important reasons is that reproductive health education in Serbia has not been systematically resolved yet, which is why young people and their peers' frequent sources of sexual health information are related to the use of the Internet and other means of public information (21).

In our study, the medical students were able to list more STIs in comparison with the other group of participants (four STIs−29.1 vs. 13.4%), who could not list any of the STIs given or could list just one STI (one STI−6.2 vs. 14.0%). It was also demonstrated due to the study conducted among young people in Nigeria (four STIs 2.8% and just 1 STI 80.2%) (23). The study conducted in Brazil on 447 students showed that the most common answers when listing STIs were HIV (men 97.0% and women 96.7%) and syphilis (men 95.7% and women 96.7%). It was in accordance with the study that conducted among the students in India, who also listed the following STIs: HIV (73.8%), syphilis (11.6%), and gonorrhea (5.7%). Our students with the same educational background most commonly provided the following answers: chlamydia (87.3%), gonorrhea (74.0%), and candida (96.4%) (24, 25). Another study was conducted among health science students in North West Ethiopia, and it was reported that 63.8% of the students had good knowledge of HIV or AIDS (26). The results of the study that conducted on the group of university students in Turkey demonstrated that 83.1% of the participants responded the question “What comes first to mind with the term sexually transmitted disease” with the word “AIDS.” The most well-known names of STDs were AIDS (94%), hepatitis (82.9%), gonorrhea (65.1%), and syphilis (61%). The least-known names have been determined as genital warts (HPV) (29.4%), genital herpes (6%), chlamydia, and trichomonas vaginalis (3.7%) (16). The results of the study that conducted in the city of Messina (Sicily region, Southern Italy) and based on the sample of 1,228 students demonstrated that only 7.9% of students were able to recognize all STIs from a list of diseases. The concern was also supported by the fact that 30% of the interviewed students were not able to properly distinguish between preventive methods and contraceptive methods. HIV or AIDS was the best-known STI among those listed; nevertheless, the question with the highest percentage of wrong answers regarded HIV or AIDS and they did not know the difference between HIV and AIDS (27).

Both student groups in our research, (78.6 vs. 77.5%) were familiar with the fact that STIs could be transferred while sharing needles, syringes, or other drug-injection equipment. The results obtained from our study were in accordance with the study that performed at the universities in Malaysia, including the groups of medical and nonmedical students, who also presented higher levels on the scale of positive responses (68.1%) (15). The study that conducted based on the sample of 246 students enrolled at the Faculty of Health Science in Turkey indicated that students had good knowledge of HIV or AIDS (61%), which means that the majority of students (81%) believed that HIV or AIDS could be transmitted *via* sharing infected syringes and needles (86%) and also from having unprotected sex with someone who had HIV. In addition, the results of the same study that conducted in Turkey proved that social work department students (75.4%) and nursing department students (64.3%) had better knowledge of HIV or AIDS and patients suffered from AIDS compared to health management students (28).

In our study, participants with both medical and nonmedical education backgrounds showed low levels of knowledge in relation to vaccination and prevention of STIs, but the group of medical students showed higher levels of knowledge (26.0 vs. 17.0%). The medical students in India had more extensive knowledge in this particular field in comparison with our medical students (85 vs. 26%) (29). Furthermore, in the Indian study, 85% of the students surveyed believed that all cases of HPV infection progressed to cervical cancer, unlike our students, because only a small number (48.2%) of them knew about this correlation. Our participants also evidenced a very limited knowledge of STIs, which was in accordance with the fact that the Republic of Serbia had the highest incidence of cervical cancer in Europe in 2002, whereas in 2012, this cancer was the fourth most common cancer in women and the third leading cause of cancer-related death in Europe (30, 31).

On average, both groups of students in our research had their first sexual experience when they became of lawful age (18 years old), which was also the case with group of nonmedical students in Turkey (28) and medical students in Central Serbia (32). However, the Turkish research indicated that medical students started being engaged in sexual relations a few years later (age of 20–25 years, 49.2 vs. 11.2%) (16). According to the National Health and Morbidity Survey (Malaysia), men (35%) and women (27%) already had sex before the age of 14 years. On the other hand, there was another report showing that 8.3% of students had already had sex with the mean age of their first sexual encounter at 15 years. Additionally, a study that conducted in China showed that women who had first debut to sex at the age younger than 18 years were more likely to have multiple sex partners compared to those who started at the age of 19 years or older (33).

The medical students surveyed in our research had a higher total number of partners [2 (1–5) vs. 1 (1–3)] and more frequent sexual relations, 2–3 times a week (45.0 vs. 39.3%), whereas the students of technical and natural sciences (more than 5 partners −32.2%) at the university in Turkey reported more partners than their colleagues studying medical sciences (more than 5 partners−17.7%) (14, 34). In one Asian study, 10.8% of the participants reported having at least one sexual partner in the past 12 months. The proportion of participants who reported having one and two or more sexual partners in the past 12 months was 8.3 and 2.5%, respectively. The highest proportion of students having two or more sexual partners in the past 12 months was found in Laos (3.8%), Thailand (4.0%), and Singapore (4.3%) (10). One of the reasons for engaging in sexual activity more frequently with different partners could be justified by the fact that university students come from various regions and towns with different cultures and values, along with the fact that while being surrounded with their new friends and classmates, they may become vulnerable and susceptible to new social and environmental influences, since they live far away from their families or relatives.

In our study, 19.7% of the medical students never used a condom during oral intercourse. Both groups of our students listed condoms (43.9 vs. 47.1%), birth control pills (12.1 vs. 6.2%), and the pull-out method (15.9 vs. 7.8%) as the first three contraception methods, which was also the case with the students from Croatia (65.7, 8.6, and 13.9%) (35).

The research conducted among the medical students at the University of Mostar in Bosnia and Herzegovina showed that 90.3% of the students used condoms during sexual intercourse, unlike our young participants who were medical students and who used condoms as contraception measures (43.9%) (36). The results of the study involving the female students of the Faculty of Medical Sciences, University of Kragujevac, Serbia, indicated that approximately 31.7% of subjects did not use any kind of protection during their first sexual debut and that about 65.5% did not use any kind of protection during their last sexual intercourse (32).

The results of the research that conducted in Croatia (65.7%) (35), Albania (51%), and Sicily (58%) among university students showed a significantly higher percent of condom use during sexual intercourse compared to the results obtained in our study (20).

Opposite to our results, the study that conducted at the University of Health Professions in Palermo indicated that the majority of students of both sexes (92%) did not use condoms during oral intercourse and were not aware of the fact that such sexual contacts presented a potential risk for the occurrence of STIs (37).

Our participants in both groups agreed that the most common reason for not using a condom was the fact that they trusted their partners (43.2 vs. 42.6%). In addition, a certain number of students from Ghana agreed with them (44.8%), whereas the other portion of students from Ghana justified not using condoms by saying they were being shy when purchasing them (24.9%) and also by stating that they did not like using it because it reduced the sense of pleasure during sexual intercourse (17.5%) (38). The other reasons were related to the feelings of invincibility, trust based on the appearance or relationship quality, and desire to live in the moment (10). An increasing number of youth population in the Republic of Serbia tends to follow the modern sexual behavior tendencies, whereas it is only a small number of them who use efficient contraceptive methods. Even if they use a condom at the beginning of their sexual activity, the majority of adolescents continue to be using the traditional and insufficiently effective pull-out method (*coitus interruptus*). Feeling too ashamed of reaching out for the contraceptive methods, overwhelmed with fear of going to the doctor's office, complete with the fear that their social environment will find out the fact that they are being sexually active are the main reasons for such a condition, apart from insufficient knowledge and all the prejudices related to the contraception use (2, 39). On the other hand, the economic aspect presents a significant factor for not using contraceptive means as well.

Medical students underwent preventive gynecological or urological examinations, unlike nonmedical students (51.2 vs. 35.5%), which could be associated with more knowledge on STIs. This also happened to be in line with the conclusion given in the study conducted among employed medical professionals in Saudi Arabia, where 68.5% of the participants reported to have undergone preventive gynecological or urological examinations (40). The results of the study that conducted in Central Serbia, which included the group of medical (female) students, demonstrated a slightly lower percent of the abovementioned preventive examinations. In addition, the study showed that 63.6% of the selected population had never performed a Pap test. Moreover, the results indicated that 23.1% had never found out their Pap test results (32).

On the other hand, the prevalence of STIs was remarkably low in some Asian countries. Regarding sexual behaviors by country, the proportion of students who reported having been diagnosed with an STI in the past 12 months was 0.0% in the Philippines and Vietnam, 0.1% in Malaysia, 0.2% in Thailand, 0.4% in Indonesia, Myanmar, and Singapore, 0.7% in Cambodia, and 1.5% in Laos (10).

The primary limitation of this study was its cross-sectional design which did not enable any inferences about potential causal relations between the explanatory variables. Furthermore, self-reporting nature of the questionnaires also presented a limitation of its own. Finally, our sample included a group of students attending only one University. Taking into consideration the fact that other universities were excluded, the generalizations were necessarily limited.

## Conclusion

The aim of this study was to describe the STI knowledge, sexual habits, and risky sexual behavior in relation to STIs among medical and nonmedical students at the higher education institutions in Belgrade, Serbia. Due to the findings presented in the paper, we concluded that it was highly necessary to evaluate sexual health education of students. Moreover, we concluded that there was still a gap of knowledge pertaining to the STIs and their preventive measures, excluding HIV infection that is generally well-known. Appropriate sexual health education administered by health professionals should provide students with all the necessary knowledge, instead of allowing them to collect data while browsing the Internet and other social media as well. Taking into consideration the results of our study and the number of women suffering from cervical cancer on the territory of the Republic of Serbia, we reached the conclusion that the level of awareness should be increased, and that reproductive health education should emphasize the importance of regular screening and administering HPV vaccines as a preventive measure.

## Data Availability Statement

The original contributions presented in the study are included in the article/supplementary material, further inquiries can be directed to the corresponding author/s.

## Ethics Statement

The studies involving human participants were reviewed and approved by Ethics Committee of Faculty of Medical Sciences, University of Kragujevac, Serbia. The patients/participants provided their written informed consent to participate in this study.

## Author Contributions

SS, IS, and VV contributed to the design and writing of the manuscript. SD, SRade, and SRado conducted the statistical analyses, commented on the manuscript, and contributed to the background and discussion section. DR, KB, JA, and JT contributed to the investigation. All authors reviewed the results and approved the final version of the manuscript.

## Conflict of Interest

The authors declare that the research was conducted in the absence of any commercial or financial relationships that could be construed as a potential conflict of interest.

## Publisher's Note

All claims expressed in this article are solely those of the authors and do not necessarily represent those of their affiliated organizations, or those of the publisher, the editors and the reviewers. Any product that may be evaluated in this article, or claim that may be made by its manufacturer, is not guaranteed or endorsed by the publisher.
